# “Do You Understand What I Am Saying?”: Exploring Language Practices in Describing Life With Diabetes

**DOI:** 10.1177/23333936261426775

**Published:** 2026-03-02

**Authors:** Jessica Gonzalez, Renee Crossman, Jude Spiers

**Affiliations:** 1 University of Alberta, Canada; 2 Memorial University of Newfoundland, St. John’s, Canada

**Keywords:** diabetes management, diabetes, language analysis, language practices, insulin pumps, self-identity, Canada

## Abstract

Language is a vehicle to express and defend one’s desired identity by employing socially normative strategies to promote and protect a preferred identity. In this qualitative secondary analysis, we explored the language practices in 30 interviews with 15 adults with diabetes who use insulin pumps. Inductive thematic analysis guided by Identity Process Theory illuminated how participants expressed and defended their preferred identities. Language strategies such as personification, humour, sarcasm, metaphors, similes, co-constructed stories, and joking, indicated trust with the listener and the speaker’s association with diabetes. Metaphor and similes provided a comprehensible analogy to articulate experience. Distancing strategies, such as objectification and sarcasm, reflected a sense of ownership of diabetes. Protecting self-esteem and self-efficacy through whispering and justification was important when describing diabetes practices as deviant or individualized. Clinicians’ attendance to language practices gives insight into identity and identity threats in a way direct questioning cannot.

## Background

Diabetes Mellitus (DM) refers to the body’s inability to produce or use insulin effectively to regulate blood glucose levels, resulting from the loss of insulin-producing cells or acquired insulin resistance. Individuals with type 1 diabetes (T1D) need to administer insulin through syringes, insulin pens, or an insulin pump, as do individuals with type 2 diabetes (T2D) if oral or other injectable medications are insufficient to manage blood glucose levels (Hannon, 2017; Padhi et al., 2020). Continuous Subcutaneous Insulin Infusion (CSII), or insulin pump therapy, is a method of managing diabetes by administering insulin throughout the day at a set basal rate and intermittent boluses to maintain blood glucose levels (Haddadi et al., 2021). Current standards for diabetes management include counting carbohydrates, monitoring blood glucose levels, engaging in regular physical activity, making dietary adjustments, and maintaining a glycosylated hemoglobin (HbA1c) level of less than 7% (American Diabetes Association, 2025; Diabetes Canada, 2025). These best practice standards for managing diabetes aim to prevent short and long-term complications. However, 34% to 64% of people living with diabetes (PLWD) do not follow their self-management plan as prescribed by healthcare professionals (HCPs) (Perrin et al., 2017; Polonsky & Henry, 2016). Contemporary guidelines do not always accommodate the contextual factors associated with diabetes management, such as fear of hypoglycemia, stereotypes, fear of gaining weight from insulin, the time required to manage diabetes, forgetting to administer insulin, socioeconomic status, social situations that may preclude administering insulin, and a poor relationship with diabetes health care providers (Brundisini et al., 2015; Walker, 2022).

Diabetes management is unique to each individual and embedded in every aspect of life, physically, psychologically, and socially (Callahan Fagan & Parsons, 2021; Walker & Litchman, 2021). How an individual relates to their diabetes influences how they self-manage. A negative psychological relationship with diabetes can contribute to distress, burnout, depression, anxiety, and non-adherence (Clausi & Schneider, 2017; Due-Christensen et al., 2018; Perrin et al., 2017; Skinner et al., 2020; Walker, 2022). Diabetes management comes with individualized challenges, and some have referred to it as a full-time job (Rogvi et al., 2021). Diabetes does not exist in a social vacuum (Abdoli et al., 2020), and PLWD must integrate their diabetes into the unpredictable and sporadic aspects of life; therefore, research needs to counter the notion of easy control (Vallis, 2015; Walker, 2022).

### The Purpose of Language in Diabetes Management

Individuals use language to communicate experiences, emotions, and perspectives. People living with chronic illnesses employ metaphors, similes, and humor as linguistic tools to convey the dimensions of their experience with their condition (Bullo, 2020; Chircop & Scerri, 2018; Delaney et al., 2020; Munday et al., 2020; Sinnenberg et al., 2018). For example, humor may serve to mask serious messages, cope with anxiety-inducing experiences, and maintain social composure (Schöpf et al., 2017). Those with chronic illnesses, including PLWD, utilize in-group language practices to foster understanding and create safe conversational spaces (Bock, 2022).

Similar to others who live with chronic illness, PLWD use metaphors and describe diabetes as an object, as a sinister actor, the body as a machine, diabetes as a war to win, and convey social norms (Sen & Merzali Celikoglu, 2019; Sinnenberg et al., 2018; Youngson et al., 2015). Using a micro-lens offers insight into how PLWD use language to reflect the realities of life with diabetes and how their perception of diabetes is intertwined with their sense of personal and social identities (Breakwell, 2010; Dunning et al., 2017). Such language practices offer insights into individuals’ understanding and management of their condition (Bullo, 2020; Chircop & Scerri, 2018; Costabile et al., 2020; Delaney et al., 2020; Munday et al., 2020; Sinnenberg et al., 2018). However, limited research exists on how PLWD perceive and express their identities in relation to diabetes or their strategies for preserving their preferred identities.

## Objective/Research Question

In this secondary analysis we aimed to explore how PLWD who use an insulin pump employ language practices to express their self-identity in terms of diabetes and respond to challenges to that identity. To interpret our inductive thematic findings, we used Identity Process Theory (IPT), a combinational model of identity construction, threat, and coping (Breakwell, 2010). The original study was a blended focused ethnography and critical discourse analysis design to explore the enactment of diabetes by those who use insulin pumps. The research question in this secondary analysis study was *“how do PLWD who use insulin pumps employ language practices to express their experiences with diabetes, their sense of self-identity, and strategies to cope with threats to that preferred identity”?*

## Research Design

This study involved a secondary analysis of 30 interviews conducted as part of a larger qualitative study, described below. The Principal Investigator (PI: RC) of the original study, who lives with T1D and uses an insulin pump, generated data rich in-group language referring to self-identity in relation to diabetes and the impact of external influences. The secondary analysis study capitalized on the data’s relevance to the PI’s primary research and utilized the entire raw dataset (Delaney et al., 2020). The secondary study’s team included the original study PI, the secondary study PI (JG), who lives with T2D and uses insulin injections, and a qualitative expert (JS) in linguistic analysis and diabetes research without personal experience of diabetes.

The original research was a blended focused ethnographic and critical discourse analysis of diabetes practices from the perspective of PLWD who use insulin pumps. The PI sought not only a description of what individuals were “doing” in the context of diabetes self-management, but also how the intricate relationships and resources align or collide in this process of enactment (Barton, 2008; Cruz & Higginbottom, 2013; Knoblauch, 2005; Pink & Morgan, 2013; Wall, 2015). The research question was: How do people with Type 1 diabetes who utilize insulin pumps enact diabetes? Drawing on Mol (2002, 2008), enactment was conceptualized as the interactive and iterative practices of problem-solving, decision-making, and sense-making by participants as they managed their diabetes.

Overall, the original research demonstrated that diabetes enactment includes the practices of decision-making, problem-solving, and sense-making; these practices exist in networks, and that “good, competent, self-management” practices are discursively conceptualized and constructed. Shifting power relations influence understanding of “correct” knowledge at the time and have implications for individuals living with diabetes, family, and friends, as well as the health care providers who support them. Based on the findings of the original study, the aim of this secondary analysis was to explore language practices used by these participants in their interviews and illustrate how they co-constructed knowledge and the impact of diabetes on their self-identity.

We conducted a qualitative thematic analysis in two analytic phases: a descriptive phase to examine language’s role in reflecting experience and self-identity, encompassing paralinguistic cues, words, phrases, and stories, which were then interpreted in phase two using the lens of IPT. Both the parent and current studies received ethical approval from the University of Alberta and Newfoundland and Labrador Health Research Ethics Authority (PRO#00081587; HREB #2018.113). Confidentiality was maintained by the removal of personal identifiers and any potentially identifying contextual information.

### Theoretical Lens: Identity Process Theory

Self-identity refers to how one views oneself and understands who one is (Caplan et al., 2016). Identity Process Theory (IPT) encapsulates the malleability of self-identity and how it adapts to the dynamic nature of an individual’s environment. According to IPT, identity consists of *content* and *value* dimensions. The *content* dimension refers to the characteristics believed to define oneself, such as group memberships, social labels, values, and attitudes (Bardi et al., 2014; Hauge, 2007). The *value* dimension reflects the positive or negative value attributed to each content component based on social beliefs and personal ideals, continually evolving throughout the lifespan (Breakwell, 2010, 2020; Hauge, 2007).

The socio-psychological interactional processes underlying identity construction can be illuminated by examining how individuals cope with changes to their self-identity. IPT suggests individuals strive for desirable states of identity, referred to as “identity motives.” There are seven identity-motives: *continuity* across past, present, and future identities, *distinctiveness* from others, *self-efficacy*, *self-esteem*, *belonging* or acceptance by others, finding *meaning* in life, and psychological *coherence* (Bardi et al., 2014).   Identity motives are satisfied when individuals are comfortable in their current chosen identity. IPT postulates that when the social context challenges these identity motives, coping strategies are used to minimize threats at the intra- and inter-personal and group/inter-group levels (Bardi et al., 2014; Breakwell, 2010). Researchers have used IPT to explore how identity changes with aging (Sneed & Whitbourne, 2005), strokes (Walder & Molineux, 2017), sexuality (Jaspal, 2021) and health risks (Breakwell, 2020).

There is a paucity of research exploring the connection between IPT and identities in diabetes (Beatty & McGonagle, 2018). Daily, PLWD self-reflect on their experiences and interactions, choosing which experiences they will accommodate and assimilate into their diabetes-specific identity (Beatty & McGonagle, 2018). PLWD continually explore, adopt, and reject ways of understanding themselves as subject to stereotypical rules about diabetes self-management (Walker, 2022), and will use coping strategies when aspects of their identity are threatened (Beatty & McGonagle, 2018). Our guiding assumption in this study was that the research participants would employ specific language practices to express their identity motives and cue when they were threatened.

### Sampling and Recruitment

The original study setting was a province in Atlantic Canada. The purposive sample consisted of 15 English speaking participants who were 18 years of age, had either Type 1 or 2 diabetes, and used an insulin pump to manage their diabetes. Recruitment occurred through community posters and social media posts by the Diabetes Canada regional office. Participants provided written informed consent. The sample consisted of nine individuals who identified as women and six identifying as men, ranging in age from 26 to 70 years old (mean: 47 years). Eleven participants lived with T1D, and 4 lived with T2D, with 11 participants residing in urban areas and 6 in rural areas. All participants described having some form of post-secondary education. The average time since diagnosis was 27 years, and the average time using an insulin pump was 10 years. Three participants lived alone, and the other 12 lived with at least 1 other person. Eight participants regularly received diabetes education, and seven participants did not. Each participant was interviewed twice, resulting in a database of 30 interviews.

### Data Generation and Analysis

Data for the primary study were generated from August 2018 to February 2020. Participants’ first interviews were approximately 50 to 70 min, and second interviews were 40 to 60 min. Topics explored included: the diagnosis story and technology employed to administer insulin, daily self-management activities, the use (or not) of resources, tailoring of generic guidelines, dealing with stigma, and when blood glucose is “not on target.” All data were digitally recorded and transcribed verbatim.

In this secondary analysis, all data were initially analyzed using inductive reflexive thematic analysis (Braun & Clarke, 2022). In phase one, we focused on descriptive analysis. First, we repeatedly listened to recordings and read transcripts to facilitate familiarity with the data. Then, we generated initial open codes, and as analysis proceeded, we searched for, iteratively reviewed, and refined potential themes until settling on the themes that accounted for the dataset. Moving to phase two, within these themes, we identified lexical units (single words or phrases) and determined their linguistic nature and function in communicating an idea using IPT to determine which concepts best matched our inductive themes (Bouncken et al., 2021).

Weekly team meetings allowed us to discuss ongoing analysis and interpretation which was important as the original researcher was able to clarify the context of each interview. We group coded the data, led by the PI for this secondary analysis study. The parent study PI was key for any questions or initial interpretations of the original interviews. Any discrepancies in data interpretation were discussed until consensus was reached. Analytic redundancy was achieved thematically in the larger study by the 15th participant, but no assumptions about saturation were made in the current study. We used recommendations by Ruggiano and Perry (2019) to determine that secondary analysis was suitable given the relationship between the parent study and the intended secondary analysis, inclusion of the original study PI, determining how the secondary analysis was related to, but distinct from, the primary analysis, and ensuring ethical standards of study approval and protection of participant confidentiality in the secondary analysis. We started with uncoded transcripts and audio recordings. We used verification strategies to incrementally check for threats to rigor and to make analytic decisions (Morse et al., 2002) and we kept memos and reflective notes throughout the analysis. Once potential themes in the data were identified, Identity Process Theory (IPT) guided the interpretation of meaning and significance in terms of expressed and perceived identity.

## Results

Each participant described their experiences with diabetes and their self-identity through distinct language practices and common terms utilized in the diabetes community. The themes from the inductive thematic analysis were: (1). Creating a Bridge of Communication to Strengthen the Relationship; (2). Am I Comfortable With You or Not? (3). Associating With and Distancing from Diabetes and; (4). How I Express Importance During a Conversation. Within these themes, specific language strategies were identified and described.

### Creating a Bridge of Communication to Strengthen the Relationship

Participants universally discussed the challenges of diabetes management and widely used metaphors (Delaney et al., 2020). A common metaphor was a bridge, which represents the communication pathway between a person living with diabetes and a person without diabetes. When the bridge is fragile, participants felt people without diabetes expressed judgment due to their difficulty understanding and appreciating their diabetes journey. When the bridge erodes, PLWD lose trust in the interaction and are less willing to discuss their diabetes experiences. However, to mend the bridge or to facilitate better understanding and build empathy, participants strategically incorporated specific language practices such as rhetorical questions, similes, and metaphors, which acted as invitations to understand an individual’s journey with diabetes. In the examples below, the specific language practices are italicized.

#### Rhetorical Questions

Rhetorical questions engage the listener by presenting an answer that places the listener in an active role in the scenario. In the example, below, the participant uses a rhetorical question (italicized) to claim the hardest part of living with diabetes are the “lows” or hypoglycemia, allowing the listener to analyze and empathize. The participant spoke quickly in the present tense with a little pause as she recounted how and why she forms priorities, which creates a sense of urgency:

*The hardest part is the lows, right?* . . .You’re in the park, you just got there, it’s 10:30, your meal’s coming up and you’re low, so you got a toddler running, one could be crying, now that I got two [children], and here you are low, so, like, you can’t go; you just gotta stop, and you gotta try to explain to a 3-year-old, like, ‘You gotta stop for a second’, and you gotta sit and have something, and then you gotta wait for it to work, and sometimes you’re fine. . . . sometimes the Dexcom comes off when I’m not symptomatic, so those times, like, okay, I’ll have my candy and then I’ll go on, but other times you can’t even see straight, so that’s the hard times. . . [Participant 10]

#### Similes

Similes facilitate understanding of diabetes-specific tasks, for example, injections, by explicitly equating them to a common activity, such as brushing teeth (italicized below). Similes help the listener empathize, strengthening the relationship.

It is, to me, yeah, I always compare it, when people ask, you know, they used to say, ‘You know, I can’t believe you’re taking needles. *How do you take needles every day?’. Well, it’s the same, for me, as brushing my teeth, except I do it before meals instead of after. I don’t look at it any differently.* [Participant 2]

#### Metaphors

The participant in the extract below expresses frustration when those without diabetes believe it is supportive to boldly inquire whether the participant’s blood glucose is “normal.” This is often perceived as intrusive and an explicit or implicit criticism of self-management. To emphasize the intrusive nature of this inquiry, the participant utilizes a metaphor to compare the question “What is your sugar?” to asking someone if they “pooped today.” This direct comparison forces a realization that diabetes management is a private and intimate activity, and that unwanted feedback and questions can be regarded as embarrassing, insulting, and offensive.

As an example of our analytic process, this excerpt below was initially coded as “frustration with bodily function on display.” Our subsequent analysis focused on deciphering the purpose of the language practices, metaphors (italicized), in this case, during participants’ dialog. We selected the IPT motive that best encapsulated each theme. The theme Creating a Bridge of Communication to Strengthen the Relationship aligned with the IPT motive of self-esteem, as fostering mutual understanding and trust directly contributes to participants’ comfort and confidence, thereby enhancing their self-esteem regarding their diabetes experience.

But the other thing is too it’s such a – like, we don’t go around asking people – okay, so I’ll put it this way – so, what is your sugar, right? *Sugar is – it’s an internal process; it’s an internal bodily process. Like, we don’t ask people if they’ve pooped today.* [Participant 1]

### Am I Comfortable With You or Not?

Participants used specific language practices to indicate if they felt accepted as an individual with diabetes. Language practices reflecting comfort and acceptance included co-constructed communication, joking, laughter, and sarcasm.

#### Co-Constructed Communication

Co-constructed communication occurs when a person starts a topic of conversation, and the other person continues the same topic (Van Bramer, 2003). In this extract, the speaker and the listener spoke about how natural diabetes management is at this point in their diabetes journey. The continuing conversation and back-to-back responses (italicized) exemplify that each is comfortable:

Participant 9: *I have a backup for my back up, all the time, right? But I never thought of it as being a challenge. I guess it’s because I’m so used to doing it now, right?*

Interviewer: *Yeah, it’s almost like – one lady explained it to me about, you know, checking her sugar and having stuff with her. She said somebody asked her about checking her sugar before she ate, and she said, ‘Well, I look at it like this – I look at it like brushing my teeth, only it’s something I do before I eat instead of after.’ So she said, ‘Do you - ‘So she says – kinda gave it right back to me. She said, ‘Do you consider brushing your teeth or washing your face something – you know, with respect to planning stuff?’ and I said, ‘No, not really’, and she said, ‘Well, that’s how I look at my diabetes planning.’*

Participant 9: *Yeah.*

Interviewer: *She said, ‘It’s like it’s just something that’s so ingrained in what I do every day that I don’t even think of it anymore,’ you know, so like that.*

#### Laughter and Joking

Laughter and joking express comfort and enjoyment during a conversation (Schöpf et al., 2017). In the example below, the topic is the unpredictable nature of daily blood glucose. The speaker’s joking (italicized) indicates they are comfortable talking about this experience and recognizes that the listener will understand the inside joke.

. . .there’re some days where I feel like my diabetes is very well balanced, and I’ll even go into the lunchroom and say *‘Get the Nobel prize ready because I think I’ve just cured type 1 diabetes’. . .* [Interviewer]

#### Sarcasm

Sarcasm, or the use of irony to mock or convey contempt, often manifests in the utterance’s context and tone of voice (Suzuki et al., 2017). In the extract below, a participant’s partner retells a conversation with her husband’s physician about her role in helping her husband self-manage. She expresses frustration that her support and help are perceived as neither acknowledged nor appreciated by the physician, but did not directly say this, although she expects the listener to gradually catch on as they piece together the sarcastic verbal and non-verbal (italicized) cues.

‘Cause she [physician] said in the first meeting, she said something about, you know, ‘That’s not your role, really. You’re his partner, and you need to do, you know, like fun stuff, partner stuff.’ I said, ‘Well, we do that.’ I said, ‘That’s not an issue.’ It’s not that *I’m working my fingers to the bone taking care of his diabetes and there’s no fun involved. I said, ‘No, we do that’, but I said, ‘I just feel that it’s a help for him. I don’t mind doing it. What the hell else am I gonna do? Knit?’, you know what I mean? I get enough time at that, so I said, ‘There’s no problem with it’, so she kinda- sorta- after a while, sort of, you know, figured out where I was coming from* [Spouse of Participant 8]

In contrast, when PLWD felt uncomfortable, they used specific language practices that illustrated how they expressed uncertainty about the safety of self-expression, often demonstrating their perceived secretive or subversive self-management practices. These language practices include whispering, justification, filler words, and pauses.

#### Whispering

PLWD whispered during conversation to express discomfort when talking about activities perceived as deviant. In the following example, the speaker whispered as they recounted how they might decide what to eat when in a restaurant, eventually avoiding revealing their potential choice in terms of sweet foods, instead referencing (italicized) conventionally safer savory foods high in fats and proteins:

Like, if you got a piece of pie and a piece of cake, it’s better to eat the cake than the pie because of the fat content in the pie, it takes your body long, well to break it down, whereas the cake’s a simple sugar. So, now, say if we went out to supper tonight, and you said, ‘Let’s have the dessert’, *well I might have a little piece of cake* or, like, if I went out for supper, I would probably choose – I dunno, I wouldn’t have a plate of fish and chips. I would probably have, I dunno, some chicken and . . .. [Participant 11]

#### Justification

All participants individualized their self-management recommendations from health care providers. Ideal diabetes standards are acknowledged, but their individualization was justified, often to resist perceived social criticism. In the excerpt below, the speaker noted that, according to dietary advice, Chinese food can be challenging. The speaker endeavors to validate their choice to have Chinese food if they desire, as diabetes does not limit *what* one can eat, as more insulin can balance the insulin/carbohydrate ratio. The use of “just” (italicized) alerts the listener that the speaker’s choice is significant, and the listener’s interjection and echoing of her words reflect the synergistic agreement.

Interviewer: It’s not that I can’t eat the Chinese food; it’s *just* that it’s my choice to not have to negotiate, and like you said, be all day.

Participant 2: Exactly.

Interviewer: Trying to deal with the sugars ‘cause, to me, it’s not worth it.

Participant 2: It’s not worth it - it’s not worth it to me at all.

#### Fillers and Pauses

Pauses give the speaker time to choose how to express themselves, create a dramatic effect, or signal importance. Fillers, such as repeating the same word or sighing (italicized), give the speaker time to compose their speech and to express emotional burdens. For example, one otherwise confident and well-spoken participant employed hesitancy, stuttering, and pauses solely when talking about a socially sensitive topic, their weight management, as weight is stigmatized not only in the diabetes community but in society in general (Himmelstein & Puhl, 2021).

*. . .I, I-* really get upset with *the- the* weight thing, and there’s nothing I can do about it [*pause*] and *uh* [*long sigh/pause] I- I*, want to eat what I’m eating because I enjoy eating what I eat, *um uh [pause]* and but I know that it’s not gonna help with the weight *[long pause].* [Participant 8]

### Associating With and Distancing From Diabetes

Participants showed how they both “owned” diabetes and attempted to disassociate from it. Associating with diabetes was exemplified when the speaker employed possessive adjectives, such as “my,” or possessive nouns, such as “mine,” to describe ownership of diabetes experiences. Possessive language strategies were noticeable when participants talked about what they had learned or competencies in a particular domain. Marking diabetes in first-person possessive terms, such as, “my diabetes, my sugars, my pump” or naming their pump, reflected the individual’s degree of comfort with this aspect of their self-identity motives of self-esteem, and self-efficacy. In the excerpt below, the speaker explains their Dexcom G6© (continuous glucose monitoring (CGM) system) helped maintain blood glucose within reasonable parameters. The speaker had a high degree of comfort and confidence with their diabetes management and specifically “owning” sugar levels (italicized) reflected this competence:

. . . I have a Dexcom now. I didn’t have it a year ago and *manage my* sugars a lot tighter. So tight that the diabetes educator says I need to relax and give myself some credit instead of beating up on myself, but things are improving – much better – much, much better, s*o my sugars are more in range*, and things are just, all in all, going a lot better. [Participant 5]

When participants conversationally distanced themselves from their diabetes, they used specific objectified terms to describe their diabetes, such as “the,” “thing,” or “it.” These terms exemplify a lack of specificity and possession, creating a distance between the individual and their diabetes. In the following conversation about unexplained high blood glucose, note how the speaker moves between association and dissociation and distancing language (italicized) when their action was unsuccessful. Distancing mitigated the threat to their sense of self management competency:

Interviewer: You said normally you go up to 15 or 16, and you know why, so now you’re looking at a 19.8 and you don’t know. What’s the first thing you did?

Participant 9: I changed my site. The first thing I did was I changed my site because there must be something malfunctioning. Okay, so now *the site i*s changed, still *the sugars* are still up higher. Then I took out *the insulin* that was there because I figured it was tainted or whatever the word you want to call it – damaged. *Got a new one, put that in*, and did all the process. *I didn’t do both together* because I needed to know what it was.

### How I Express Importance During a Conversation

Participants expressed significance of ideas or experiences through distinct language practices, such as repetition, hyperboles, elevated tone, and anthropomorphism. Repeating a word two or more times was a common way to draw attention. In this example, the critical importance of always carrying glucose tablets (italicized), is highlighted:

Repetition:

I have those glucose tablets in my pocket. I do not go anywhere without them. A couple of times that I did, I find that it causes me a lot of anxiety. . .and actually, it was only a couple of weeks ago I . . . literally went to the end of my driveway, didn’t have it, and my sugars went low, and it could take me five minutes to get back to the top of my driveway. By the time I got back, I was like 2.5 – *really, really* low– and it’s scary. [Participant 1]

#### Hyperboles

Hyperboles were utilized when participants discussed how, despite care and attention, the same self-management practices used 1 day may not work the following day. The speaker adds extra emphasis (italicized) by exaggerating glucose fluctuations to cue the listener about the frustration associated with this experience.

Let’s just say my blood sugar is 7. I’m gonna eat a banana but I’m going for a run, so how much insulin am I gonna need? Now, today, I might take no insulin for the banana, and my blood sugar would be great. *Tomorrow, I might take no insulin for the banana, and my blood sugar would be sky high when I get back.* [Participant 2]

#### Elevated Tone

An elevated tone provides additional emphasis to a statement. In the extract below, the participant, a nurse, described choosing to have a blood glucose level above the recommended range because of the difficulty of securing a break at work. Their elevated vocal tone and volume, that is, raising their voice, is italicized, underlined, and in upper case to communicate their frustration with the situation.

Prior to the pump, I guess it was harder because I would have issues with lows, and you would take your insulin ‘cause you had just gone on your break, and then something happens on the floor and you’re back out, and before too long, *YOU’RE* the one who’s in trouble, right, so that happened a couple of times . . . it was only previous to the pump that I had any problems, and [brief pause] and you would sometimes tend to stay a little bit higher when you were working because I’d be afraid of having a low. *I DID NOT* want to be high, and that really upset me, but at the same time, the thought of having a **low** and the *EMBARRASSMENT* that came with having a low, because people don’t understand enough to know that, if *I HAVE A LOW, I JUST NEED TO TREAT IT, AND THEN I CAN MOVE ON; IT’S ALL GOOD*, but one evening when I had a low, like I was just saying, I was sent home – I wasn’t permitted to finish my shift! *[A long pause before the listener spoke]* [Participant 6]

#### Anthropomorphism

Anthropomorphism refers to attributing human attributes to an object, most commonly the insulin pump or continuous glucose monitor. Participants regarded their insulin pumps simultaneously as part of themselves and as an external, distinct, and active actor with its own agency, as this participant said

 “I can’t eat pizza. If I eat a slice of pizza, *I’m sure my pump almost hiccups.”* In this example, the image of an insulin pump “hiccupping” is both evocative and humorous, allowing the listener to appreciate the significant amounts of insulin needed to deal with pizza. [Participant 7]

## Interpretation and Discussion

Self-identity depends on a variety of aspects of an individual’s life, and for PLWD, diabetes is one such aspect, referred to as diabetes identity (Maietta, 2021; Walker & Litchman, 2021; Øversveen & Stachowski, 2023). PLWD learn to use specific language practices to express their self-identity and to withstand and mitigate threats to their sense of self-identity. Our study focused on exploring how PLWD use a combination of language practices to express or defend their identity motives: distinctiveness, self-esteem, self-efficacy, and belonging. The secondary analysis of interviews provided insight into how participants presented and defended their desired identities, belongingness, self-esteem, and self-efficacy through their language practices. Building a bridge of communication through rhetorical questions, similes, and metaphors reflected the participants’ sense of acceptance and understanding, providing different ways to understand the significance of the PLWD’s self-management practices. Laughter and sarcasm indicated a level of trust and safety in co-creating meaning with another person whom they saw as an insider-who-lives-with-diabetes, but also an unknown outsider and healthcare researcher. Participants also whispered about individualized or “deviant” management practices.

### Diabetes and Self-Identity

Persons living with T1D are more likely to report that their diabetes is a central element in their social identity than those living with T2D (Costabile et al., 2020). Conversely, Kato et al. (2016) argue that external factors, such as other people’s perspectives and opinions about T2D, are significant in determining whether diabetes is a central element of self-identity. However, Amorim et al. (2014), suggest that PLWD accept or reject their diabetes, yet our participants demonstrated they could both accept and integrate diabetes while distancing themselves from their diabetes, depending on the context and audience. Similar to the findings of Martínez-Angulo et al. (2024), the participants in this study created bridges of communication and felt a sense of belonging depending on the contextual influence of biomedicine. When participants’ self-esteem was threatened by not meeting biomedical targets for example, bridges eroded and a sense of belonging diminished. That is, language practices both reproduced or mitigated identity motives, depending on the context.

Contemporary conceptualizations of chronic illness suggest that individuals continually move between states where the condition is in the background so the individual can focus on “life” and at other times, the condition is in the foreground, dominating the individual’s attention (Kristensen et al., 2018; Paterson, 2001). Learning to live with diabetes requires self-identity evolution, as PLWD must continually balance self-management needs with current social roles and activities (Kato et al., 2016). Identity Process Theory (IPT) suggests that individuals undergo identity assimilation (manifested as maintaining self-consistency in their beliefs and practices), identity accommodation (how they adapted their self-identity for diabetes), and identity balance (in which they maintained a sense of self that could change when necessary) to shape their self-identity according to experience (Bardi et al., 2014).

### Language Practices Showing Expression of Self-Identity

**Figure 1.**
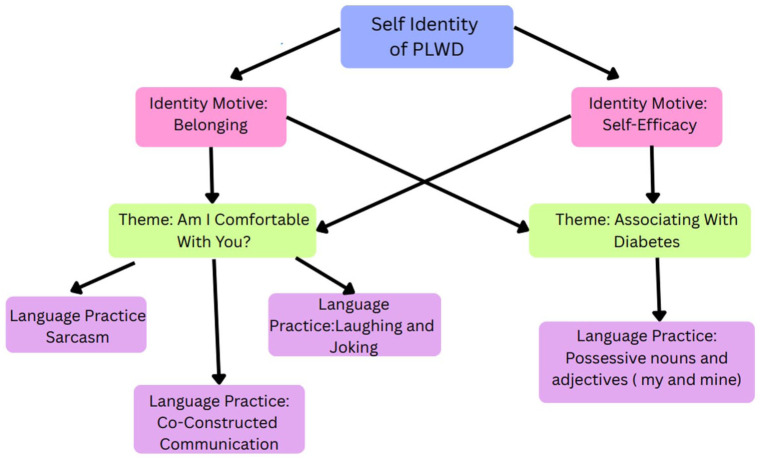
Expression of self-identity: Languages practice used when identity motives self efficacy and belonging are expressed during a conversation. The figure exemplifies the themes and the associated language practices used to express the identity motives *self-efficacy and belonging*.

In our study, two identity motives, self-efficacy and belonging, were predominant, and participants used specific language practices to show trust in the relationship and express their diabetes identity. The self-efficacy motive refers to having control and confidence in one’s current self-identity, and the belonging motive refers to a sense of closeness to and acceptance of that identity (Bardi et al., 2014). When participants felt confident in their diabetes identity, they would express the identity motives self-efficacy and belonging. To express both identity motives, participants used language practices under the themes Associating with Diabetes and Feeling Comfort, as shown in Figure 1.

Language practices such as co-constructed communication, sarcasm, joking, laughter, and the use of possessive nouns and adjectives were cues that the participant was comfortable and confident, trusted the listener, anticipated no judgment, which builds on the work of Schöpf et al. (2017) who found PLWD use different forms of humor to build relationships and mitigate the threat of serious messages. Similar to Schöpf et al. (2017), trust was strengthened if the researcher responded appropriately with humor, sarcasm, and co-created sentences. Co-construction of ideas, laughter, and sarcasm all emphasize the in-group belongingness of both people in the conversation (Schöpf et al., 2017; Øversveen & Stachowski, 2023). Like Mifsud et al. (2019), the use of possessive pronouns and adjectives to describe diabetes creates psychological ownership during the conversation. PLWD consider the discussed experience to be part of their diabetes identity, leading them to speak comfortably and confidently about that experience.

### Language Practices Showing Threats to Self-Identity

**Figure 2.**
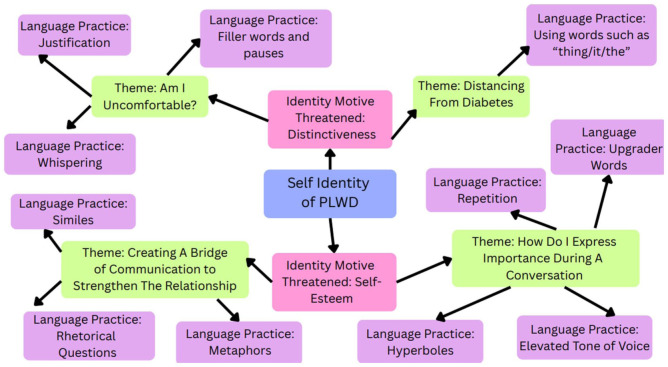
Defending self-esteem: language practices used when the identity motives self-esteem and distinctiveness are threatened during conversation. The figure above demonstrates the themes and the associated language practices used when the identity motives *self-esteem and distinctiveness* are threatened during conversation.

Our study highlighted how PLWD used specific language practices to indicate that their identity motives of self-esteem and distinctiveness were threatened. Figure 2 illustrates the language strategies used to defend these identity motives.

 Self-esteem refers to a person’s self-worth (Bardi et al., 2014). When a speaker felt that their self-managing abilities were questioned, their management did not follow medical recommendations, or they felt blamed for faults in their management, the identity motive, self-esteem, was threatened. Researchers have explored how certain words and phrases such as “non-compliant,” “poorly controlled,” and “diabetic” adversely impact self-identity (Banasiak et al., 2020; Dunning et al., 2017). Dunning et al. (2017) described how tone and hurried conversations make a PLWD uncomfortable and destroys rapport in a conversation. In our study, participants used similes, metaphors, and rhetorical questions, to build a sense of safe conversational space, as reflected in the theme Creating a Bridge of Communication to Strengthen the Relationship. When this safety was achieved, speakers were able to invite the listener to protect their diabetes identity by reflecting on common understandings and experiences, thereby improving understanding and rapport. Additionally, the participants employed repetition, elevated tone of voice, hyperbole, and up-grader words in the theme How to Express Importance During a Conversation to emphasize the parts of their diabetes identity they believe are important.

 Addressing threats to the identity motive, distinctiveness, which refers to feeling unique compared to others, was highly apparent in our study (Bardi et al., 2014). When participants felt that their experience or lifestyle with diabetes differed from others or did not follow the perceived “gold standards” of diabetes management, which could potentially invite criticism or censure, their language practices reflected both their sense of discomfort and distancing from diabetes. Participants would justify, whisper, and use filler words and pauses when they felt uncomfortable. They distanced themselves from diabetes by objectifying and referring to diabetes and resources as “thing or it” to reduce their vulnerability to being judged and criticized through hurtful comments (Franklin et al., 2018).

### Clinical Relevance: Person-Centered Care and Patientism

Healthcare systems are gradually replacing the biomedical acute model with a person-centered care model (Eklund et al., 2019). The traditional, biomedical approach’s power dynamic, passive patient roles, and checklist approach to clinical interactions can lead to patients feeling neglected and excluded from decision-making processes (Fisher et al., 2019; Liu et al., 2017; Mol, 2008; Nimmon & Stenfors-Hayes, 2016). The biomedical model holds the PLWD accountable for faults in their self-management and can exacerbate diabetes distress through inadvertent communication that misses opportunities to address PLWD’s concerns (DeMolitor et al., 2020). Strikingly, the biomedical-focused approach fails to explore ways to increase confidence in self-management and repeats irrelevant and a-contextual biomedical explanations and information (Abdoli et al., 2020; Franklin et al., 2018; Skinner et al., 2020).

Conversely, a model focusing on the person highlights the importance of Patientism, a type of activism that promotes and advocates for patients to have the right to make choices in their care, be involved with HCPs, and that HCPs take a person-inclusive clinical approach (Dickinson et al., 2017; Liu et al., 2017; Mol, 2008; Skinner et al., 2020). Patientism promotes self-efficacy and improved care outcomes (Trout et al., 2019). An approach that prioritizes the person over biomedicine requires the clinician to employ a holistic view and incorporate the PLWD’s values, preferences, and goals into the care planning, which has led to improved care (Callahan Fagan & Parsons, 2021; Eklund et al., 2019).

Analysis of language discourse is important in a person-focused model, as language plays an important role in providing insight about the PLWD’s context to incorporate in person-centered care planning. Using terms such as control, compliance, imperative (can/cannot, should/shouldn’t), regimen, rules, cheating/sneaking, diabetic, non-compliant, poorly controlled or words that threaten dire complications, can adversely impact a person’s diabetes identity and willingness to manage (Banasiak et al., 2020; Dickinson et al., 2017; Dunning et al., 2017; Speight et al., 2021). Intentional or not, when communication is perceived as condescending and belittling, it can erode the relationship between HCP and a PLWD (Skinner et al., 2020). Dunning et al. (2017) have explored how tone and hurried conversation make a PLWD uncomfortable and destroy rapport in a conversation. Simply listening to the person may be a valid measure to not only mitigate the loneliness that accompanies the experience of self-managing a chronic condition but to identify the cues that allude to priority needs and experiences (Banasiak et al., 2020; Lloyd et al., 2018; Settineri et al., 2019). Use of Identity Process Theory (IPT) is another tool in clinicians’ relational and communicative toolbox, along with approaches such as Face Work Theory, which explains how individuals in interaction cooperate to build, maintain, protect, or threaten personal dignity, honor, and respect by using politeness strategies, to mitigate challenges to patients’ sense of autonomy, belonging and competence and to maintain clinicians’ sense of professional identity (Cullinan et al., 2022; Fosgerau & Kaae, 2021; Murad et al., 2017).

Our findings highlight how PLWD use language to express their self-identity motives and how they cope with threats to these self-identity motives. Diabetes is multifaceted and complex, so HCPs need to slow down and listen to how PLWDs relate to their condition and practices, especially with the equipment that externally monitors and controls their blood glucose levels (Crossman, 2021; Franklin et al., 2018). Listening closely to the subtle cues in communication may be the key to identifying what a PLWD is telling you when they appear not to say anything. Importantly, these participants’ language practices can serve as cues for HCPs to better understand their clients’ relationships with their diabetes and to create strong therapeutic relationships that form the basis of effective self-management support (Delaney et al., 2020; Settineri et al., 2019; Van Dijk, 2018).

### Strengths and Limitations

Our study aimed to understand language strategies used to manage self-identity related to diabetes. Findings should be cautiously transferred, considering participant characteristics and socio-cultural contexts. The involvement of researchers with and without lived diabetes experience added depth to the analysis. However, the sample predominantly consisted of middle-class, educated Caucasian women using insulin pumps, limiting transferability. In this study, we did not specifically inquire about socio-economic status or questions specific to the diabetes type. Therefore, we were unable to analyze the data across diabetes type and social position/location. While we did not explicitly explore socio-economic status, all participants described some form of post-secondary education. Additionally, affordability of diabetes supplies and management in general was discussed during the interviews. The influence of sex and gender was not a focus of the larger, parent study and, subsequently, was not considered in this secondary analysis. To do so in the secondary analysis would have risked adding a priori assumptions of the data. Additionally, insulin pumps are expensive; therefore, are not broadly accessible. Given that insulin pump accessibility is closely linked to socio-economic status, we have not captured a wide range of speech in potentially marginalized and vulnerable groups or diverse ethnic and cultural groups. Hence, future research is needed to expand variation reflecting socioeconomic and ethnic differences and explore the influence of sex and gender in diabetes practices in the context of insulin pumps. Further research is also needed to explore similarities and differences with those who manage via insulin injections and other medications.

## Conclusion

Diabetes, a complex chronic illness, demands continuous attention. This secondary analysis provided insight into how participants presented and defended their desired identities in terms of belongingness, self-esteem, and self-efficacy through their socially normative language practices. Building a bridge of communication by using rhetorical questions, similes and metaphors reflected the participants’ sense of acceptance and understanding and provided different ways to understand the significance of the PLWD’s self-management practices. Laughter and sarcasm are indicative of a level of trust and safety in a conversation. Participants whispered about individualized or “deviant” management practices exemplifying discomfort. HCPs must pay attention to indirect verbal cues and recognize language practices that protect diabetes identity. This understanding aids in comprehending a person’s self-management journey, needs, and challenges. Developing the skill to hear unexpressed needs strengthens relationships and enables tailored professional support for self-management.
